# Colonoscopy findings in *CDH1* carriers from a multicenter international study

**DOI:** 10.1007/s10689-025-00466-8

**Published:** 2025-05-05

**Authors:** Arjun Chatterjee, Robert Hüneburg, Qijun Yang, Shannon Morrison, Anna Bettzüge, Tim Marwitz, Stefan Aretz, Isabel Spier, Tim Ripperger, Silke Redler, Mykyta Kachanov, Alexander E. Volk, Deepak B. Vangala, Severin Daum, Elke Holinski-Feder, Verena Steinke-Lange, Kathrin Bahlke, Christian P. Strassburg, Lady Katherine MejiaPerez, Margaret M. O’Malley, Lisa LaGuardia, David Liska, Carole Macaron, Joshua Sommovilla, Carol A. Burke, Jacob Nattermann

**Affiliations:** 1 Department of Internal Medicine, Cleveland Clinic, Cleveland, OH USA; 2 Department of Internal Medicine, University Hospital Bonn, Bonn, Germany; 3 National Center for Hereditary Tumor Syndromes, University Hospital Bonn, Bonn, Germany; 4 European Reference Network for Genetic Tumor Risk Syndromes (ERN Genturis), Nijmegen, The Netherlands; 5 Quantitative Health Sciences, Lerner Research Institute, Cleveland Clinic Foundation, Cleveland, OH USA; 6 Institute of Human Genetics, Medical Faculty, University of Bonn, Bonn, Germany; 7 Department of Human Genetics, Hannover Medical School, Hannover, Germany; 8 Institute of Human Genetics, Medical Faculty and University Hospital Düsseldorf, Heinrich-Heine-University, Düsseldorf, Germany; 9 Institute for Human Genetics, University Hospital Hamburg-Eppendorf, Hamburg, Germany; 10 Genetics, Department of Cancer Genetics, Ruhr-University Bochum, Bochum, Germany; 11 Medical Department, Division of Gastroenterology, Infectious Diseases and Rheumatology, Charité University Medicine Berlin, Campus Benjamin Franklin (CBF), Berlin, Germany; 12 MGZ - Medizinisch Genetisches Zentrum, Munich, Germany; 13 Medizinische Klinik und Poliklinik IV, Klinikum der Universität München, Munich, Germany; 14 Institute for Human Genetics, University Hospital Münster, Münster, Germany; 15 Department of Gastroenterology, Hepatology and Nutrition, Cleveland Clinic, Cleveland, OH USA; 16 Department of Colorectal Surgery, Cleveland Clinic, Cleveland, OH USA; 17 Sanford R. Weiss MD Center for Hereditary Colorectal Neoplasia, Cleveland Clinic, Cleveland, OH 44195 USA; 18 Desk A30, Department of Gastroenterology, Hepatology and Nutrition, Cleveland Clinic, 9500 Euclid Ave, Cleveland, OH 44195 USA

**Keywords:** *CDH1*, Colorectal cancer, Colon cancer, Colonoscopy, *CDH1* pathogenic variants

## Abstract

Germline (likely-)pathogenic variants (PV) in *CDH1* predispose carriers to hereditary diffuse gastric cancer and lobular breast cancer. Previous studies from the United States suggest *CDH1* variant carriers have an increased risk for adenomas or sessile serrated lesions (SSL), yet data linking *CDH1* PVs and colorectal neoplasia are scarce. We aimed to investigate colonoscopy findings in *CDH1* PVs. Adults carrying a PV/LPV in *CDH1* with ≥ 1 colonoscopy between 01/01/2004–12/31/2023 were included. Patients were sourced from the David G. Jagelman Inherited Colorectal Cancer Registries at Cleveland Clinic and the German Consortium for Familial Intestinal Cancer. 103 *CDH1* PV carriers were included. Most were female (66%) and white (93.1%). The median age at first colonoscopy was 47 years. The adenoma detection rate (ADR) was 29.4% (95% CI:19.9–41.1%) in the German cohort and 48.6% (95% CI: 33.0-64.4%) in the Cleveland cohort (*p* = 0.055) and significantly correlated with age (< 45 years, 13.6% (95% CI: 6.40–26.7%); 45–49 years, 52.4% (95% CI: 32.4–71.7%); ≥50 years, 52.6% (95% CI: 37.3–67.5%); *p* < 0.001). The ADR in Cleveland was higher than the U.S. average ADR but the difference was not statistically significant (48.6% vs. 35.6%, *p* = 0.08), and the ADR in the German cohort (29.4%) was similar to the national German average risk screening cohort (31.3% in men, *p* = 0.84; 20.1% in women, *p* = 0.08). In our screening cohort with *CDH1* PV carriers, we demonstrated an ADR of 13.5% in individuals under 45 years, similar to the ADR in patients aged 25–40 years with a family history of CRC. Overall, SSL detection rate was 9.7%. Colorectal cancer was diagnosed in 3 patients (3.2%), 2/3 with an early age of onset before the age of 50 years. This first international study provides preliminary evidence of a higher ADR in U.S. *CDH1* PV carriers compared to the general population, with a high number of adenomas detected before the age of 50. This may indicate an increased CRC risk that should be explored in larger studies.

**Supplementary Information:**

The online version contains supplementary material available at 10.1007/s10689-025-00466-8.

## Introduction

Germline pathogenic variants and likely pathogenic variants (PV) in *CDH1* predispose carriers to hereditary diffuse gastric cancer (HDGC) and lobular breast cancer. Older data indicate that carriers of *CDH1* germline PVs have a cumulative risk of developing diffuse signet ring cell gastric cancer by the age of 80 that varies from 24 to 83% in female carriers and from 37 to 70% in male carriers. According to reports, the lifetime risk of developing breast cancer for female carriers is between 39 and 42% [1–3]. However, recent findings by Ryan et al. show a lower prevalence of gastric cancer (13.9%) in both sexes and breast cancer (26.3%) among female carriers. The gastric cancer risk appears to be highly dependent on family history, with the lifetime cumulative risk of advanced gastric cancer estimated to be 10.3% for male carriers and 6.5% for female carriers [4].

The results of germline genetic testing in a single commercial laboratory in over 200,000 probands with a variety of cancers reported a significant enrichment of germline *CDH1* PV in patients with colorectal adenocarcinoma (CRC) and signet ring cell cancer when compared to a cancer-free population [5, 6]. The most common cancers in *CDH1* PV carriers included breast cancer (54.6%), gastric cancer (39.7%), and CRC (9.9%). However, a large European study showed no association between *CDH1* PVs and the occurrence of CRC [7].

Two, 85 patient studies from the United States reported adenomas and sessile serrated lesions in 41–47% of *CDH1* PV carriers undergoing colonoscopy, including 27-50% under age 45 years at time of the examination [8, 9]. Currently, there is limited evidence to understand any association between colorectal neoplasia and *CDH1* PVs and whether early CRC screening should be recommended in these patients. We investigated the colonoscopy findings in patients with *CDH1* PVs undergoing colonoscopy in an international cohort of patients.

## Methods

This is a retrospective analysis of a prospectively enrolled cohort approved by the Cleveland Clinic IRB #21-1129 and at eight centers of the German Consortium for Familial Intestinal Cancer (GCFIC; Bonn, Bochum, Düsseldorf, Hannover, Hamburg, Münster, Berlin, München; central IRB approval No. 099/15 Bonn). Adult (age ≥ 18 years) germline carriers of a PV (defined as class 4 or 5 PV) in *CDH1* with ≥ 1 colonoscopies between 01/01/2004–12/31/2023 were identified through the David G. Jagelman Inherited Colorectal Cancer Registries within the Sanford R. Weiss MD Center for Hereditary Colorectal Neoplasia at Cleveland Clinic and through local databases at the eight centers of the GCFIC and the prospective cohort of the National Center for Hereditary Tumor Syndromes (Bonn). All patients diagnosed with *CDH1* PVs who require gastric cancer surveillance are prospectively enrolled in our gastric cancer surveillance and offered screening colonoscopy. At the Cleveland Clinic, colonoscopy is recommended at age 40 or 10 years younger than the age at which family members were diagnosed with CRC. In the GCFIC, 85% of patients were from University Hospital Bonn, colonoscopy was offered at the earliest of either age 25 or after a diagnosis of a *CDH1* PV. In the other GFIC centers, patients were selected for colonoscopy based on the respective centers’ recommendations and outside of the national screening program [10]. All but two patients had a confirmed *CDH1* PV prior to undergoing colonoscopy and 2 were diagnosed with a *CDH1* PV after colonoscopy. Multi-gene panel testing was obtained, if not previously performed, in the patients with CRC. No additional germline PVs variants were detected. Diagnostic colonoscopies were defined as examinations due to symptoms such as bleeding, abdominal pain, unexplained weight loss. The medical record was used to obtain demographic characteristics, personal and family history of cancer, and colonoscopy findings. Adenoma detection rate (ADR) was defined as the number of patients with ≥ 1 adenoma during their cumulative lifetime colonoscopies exposure. Advanced adenoma (AA) was defined as adenoma ≥ 10 mm, and/or with any villous component and/or high-grade dysplasia (HGD). Advanced sessile serrated lesion (SSL) was defined as an SSL ≥ 10 mm and/or with dysplasia.

Data were described using mean and standard deviation (SD) for normal continuous variables, median and interquartile range (IQR) for non-normal continuous variables, and frequency (percentage) for categorical variables. The two-sample t-test and Wilcoxon rank sum test was used to compare continuous variables between Cleveland and Germany. The Pearson’s Chi-square test and Fisher’s exact test were used to compare the categorical variables between sites and age groups. Analysis of variance (ANOVA) and Kruskal-Wallis test were used to compare the continuous variables among three age groups. Pairwise comparisons using Bonferroni adjustment were performed to assess the differences between every two age groups. One sample proportion test and exact binomial test were used to compare ADR in our screening colonoscopy data to national averages. A sensitivity analysis was conducted, including only patients who underwent screening colonoscopies. We calculated the 95% confidence intervals (CI) for ADR estimate in each of sites and each of three age groups using screening colonoscopies. Additionally, we performed an analysis to identify differences between the findings on screening and diagnostic colonoscopies. Analyses were performed using SAS software, and a significance level of 0.05 was assumed for all tests.

## Results

### Demographic characteristics

A total of 103 *CDH1* PV carriers were included in the study. Most patients were female (66%) and Caucasian (93.1%) (Table 1). Overall, 48.5% of patients had a personal history of gastric cancer, and 22.3% had a family history of CRC. Among female carriers, 39.7% had a history of breast cancer.

**Table 1:** Demographic characteristics in patients with *CDH1* pathogenic variants undergoing colonoscopy

Factors	Overall (*N* = 103)	Germany (*N* = 68)	Cleveland (*N* = 35)	*p*-value*
Female sex, n (%)	68 (66.0)	43 (63.2)	25 (71.4)	0.41^c^
Ethnicity, n (%)				< 0.001^d^
Caucasian	95 (93.1)	68 (100.0)	27 (79.4)	
African American	3 (2.9)	0 (0.00)	3 (8.8)	
Asian	2 (2.0)	0 (0.00)	2 (5.9)	
Other	2 (2.0)	0 (0.00)	2 (5.9)	
Personal History of Gastric Cancer, n (%)	50 (48.5)	37 (54.4)	13 (37.1)	0.097^c^
Age at Gastric Cancer Diagnosis, yrs, median [Q1, Q3]	45.5	44.0	49.9	0.19^b^
median [Q1, Q3]	[33.0, 53.0]	[32.0, 53.0]	[40.1, 56.7]	
Personal History ofBreast Cancer in females, n (%)	27 (39.7)	14 (32.6)	14 (52)	0.11^c^
Age at Breast Cancer Diagnosis in females, yrs, median [Q1, Q3]	48.3 [43.1, 59.0]	46.0 [43.0, 54.0]	48.6 [43.7, 59.2]	0.55^b^
Family History CRC, n (%)	23 (22.3)	12 (17.6)	11 (31.4)	0.11^c^
Age of Family Member at CRC Diagnosis, yrs median [Q1, Q3]	61.5 [60.0, 63.0]	*Not* *available*	61.5 [60.0, 63.0]	*N/A*

Statistics presented as Median [P25, P75], N (column %)

p-values: b = Wilcoxon Rank Sum test, c = Pearson’s chi-square test, d = Fisher’s Exact test. CRC = colorectal cancer, N/A = not applicable

*Germany vs. Cleveland

The median age at the first colonoscopy was 47 years [IQR 35.0, 55.9]. Cleveland patients were older (median 49.9 years) compared to German patients (median 43 years) (*p* = 0.008, Table 2). The overall ADR was 35.9% (95% CI: 27.3–45.5%), with a higher ADR in Cleveland (48.6%, 95% CI: 33.0-64.4%) compared to Germany (29.4%, 95% CI: 19.9–41.1%) (*p* = 0.055). The median age at first adenoma detection was 55 years [IQR 47.9, 59.5]. Cleveland patients were diagnosed at a younger median age (50 years) compared to German patients (56 years). Advanced adenomas were only detected in the German cohort, involving two large tubular adenomas ≥ 10 mm and one containing high-grade dysplasia (HGD). The median age at first advanced adenoma (AA) detection was 45 years [IQR 36.0, 46.0]. The SSL detection rate was 9.7% overall, higher in Cleveland (11.4%) compared to Germany (8.8%). Of the 10 patients with SSL, 4 had advanced SSLs with a size of ≥ 10 mm without dysplasia. Colorectal cancer (CRC) was found in 3 patients (3.2%), aged 29–69 years (Table 3). Multi-gene panel testing, if not previously performed in the patients with CRC. No additional germline PVs variants were detected. The molecular features of the sigmoid cancer in the two female patients who were ages 29 and 49 years were microsatellite stabile (MSS). The *CDH1* PV was detected in the 29-year-old after her diagnosis of breast and gastric cancer.

**Table 2:** Colonoscopy findings in *CDH1* pathogenic variant carriers

Factors	Overall (*N* = 103)	Germany (*N* = 68)	Cleveland (*N* = 35)	*p*-value*
Age at 1st colonoscopy, median [Q1, Q3]	47.0 [35.0, 55.9]	43.0 [32.0, 56.0]	49.9 [46.2, 57.7]	***0.008*** ^***b***^
Age at 1st colonoscopy, n (%)				***0.020*** ^***b***^
<45 years 45–49 years ≥ 50 years	44 (42.7) 21 (20.4) 38 (36.9)	36 (52.9) 10 (14.7) 22 (32.4)	8 (22.9) 11 (31.4) 16 (45.7)	
No, of colonoscopies, median [Q1, Q3]	1.00 [1.00, 2.0]	1.00 [1.00, 2.0]	1.00 [1.00, 2.0]	0.34^b^
Number of colonoscopies, n (%)				0.71^d^
1	62 (60.2)	39 (57.4)	23 (65.7)	
2	25 (24.3)	18 (26.5)	7 (20.0)	
3	9 (8.7)	7 (10.3)	2 (5.7)	
≥4	7 (6.8)	4 (5.9)	3 (8.6)	
Years of surveillance, mean ± sd	2.0 ± 3.5	1.9 ± 3.6	2.1 ± 3.5	0.85^a1^
Indication for baseline colonoscopy, n (%)				0.21^d^
Screening Diagnostic	90 (88.2) 12 (11.8)	62 (91.2) 6 (8.8)	28 (82.4) 6 (17.6)	
Hyperplastic polyps, n (%)	13 (12.6)	10 (14.7)	3 (8.6)	0.53^d^
No. patients with non-advanced adenomas, n (%)	36 (35.0)	19 (27.9)	17 (48.6)	***0.038*** ^***c***^
Age at 1st adenoma (years), median [Q1, Q3]	53.0 [47.0, 59.0]	55.0 [46.5, 59.0]	50.0 [47.9, 59.0]	0.95^b^
Adenoma detection rate, n (%)	37 (35.9)	20 (29.4)	17 (48.6)	0.055^c^
SSL detection rate, n (%)	10 (9.7)	6 (8.8)	4 (11.4)	0.73^d^
Advanced SSL detection rate, n (%)	4 (3.9)	4 (5.9)	0 (0.00)	0.30^d^
CRC, n (%)	3 (2.9)	2 (2.9)	1 (2.9)	0.99^d^
Age at CRC (years), median [Q1, Q3]	49.0 [29.0, 69.0]	49.0 [29.0, 69.0]	49.0 [49.0, 49.0]	*N/A*

Statistics presented as Mean ± SD, Median [P25, P75], N (column %)

p-values: a1 = t-test, a2 = Satterthwaite t-test, b = Wilcoxon Rank Sum test, c = Pearson’s chi-square test, d = Fisher’s Exact test. CRC = colorectal cancer, N/A = not applicable

*Germany vs. Cleveland

**Table 3:** Features of colorectal cancer in *CDH1* pathogenic variant carriers

	Patient 1	Patient 2	Patient 3 (Cleveland)
Age at diagnosis	29	69	49
*CDH1* Variant	c.1679 C > G	c.1565 + 2dupT	c.1565 + 2dupT
Gender	Female	Male	Female
Cancer Location	Sigmoid	Cecum, rectum	Sigmoid
Tumor Stage*	Stage IIIA (T2, N1, M0)	Stage IVA (T3b, N1, M1)	Stage I (T1, N0, M0)
MSI/dMMR	MSS	Both CRCs MSI-H, pMMR	MSS
Pathology	Moderately differentiated cylindrocellular adenocarcinoma	Moderately differentiated adenocarcinoma	Moderately differentiated adenocarcinoma arising in TA-HGD
Personal history Breast or Gastric CA (age at diagnosis)	Breast (49 yrs) Gastric (51 years)	Gastric	Non-Lobular Breast (53 years)
Family Hx CRC	No	No	No

Abbreviations: CA = cancer; CRC = colorectal cancer MSI = microsatellite instable; dMMR = mismatch repair deficient; MSS = microsatellite stable; pMMR = mismatch repair proficient; TA-HGD = tubular adenoma with high grade dysplasia; * tumor stage based on AJCC 8th edition

The 69-year-old male had synchronous adenocarcinomas affecting the cecum and rectum, characterized by high microsatellite instability but proficient mismatch repair status in both CRCs. Germline testing revealed a variant of unknown significance in *MSH2* (c.1275 A > G) and a pathogenic variant in *CDH1*.

To detect an age effect, we stratified the results by age. The age groups were selected to reflect different screening practices in both countries: under 45 years, as there is no routine screening in the general population in the USA and Germany; from 45 years, as screening is recommended in the USA starting at this age, but not in Germany; and 50 years and older, where screening is recommended in both countries. Table 4 presents colonoscopy findings for 103 individuals categorized into three age groups: <45 years (44 patients), 45–49 years (21 patients), and ≥ 50 years (38 patients). The average follow-up time was 2.0 ± 3.5 years, with no significant differences between age groups (1.9 ± 3.7 years, 2.4 ± 4.1 years, and 1.9 ± 3.0 years, respectively). A significant difference in ADR was observed: the lowest ADR was in patients < 45 years (13.6%, 95% CI: 6.40–26.7%), while similar ADRs were found in patients aged 45–49 years and those ≥ 50 years (52.4%, 95% CI: 32.4–71.7% and 52.6%, 95% CI: 37.3–67.5%, respectively; *p* < 0.001). No significant differences were found between age groups for SSLs, advanced SSLs, or CRC detection.

**Table 4:** Baseline colonoscopy findings in *CDH1* by age range

Factors	Overall (*N* = 103)	< 45 years (*N* = 44)	45–49 years (*N* = 21)	≥ 50 years (*N* = 38)	*p*-value
Total number of colonoscopies, n (%)					0.90^d^
1	62 (60.2)	27 (61.4)	13 (61.9)	22 (57.9)	
2	25 (24.3)	10 (22.7)	4 (19.0)	11 (28.9)	
3	9 (8.7)	5 (11.4)	2 (9.5)	2 (5.3)	
≥4	7 (6.8)	2 (4.5)	2 (9.5)	3 (7.9)	
Years of surveillance, mean ± SD	2.0 ± 3.5	1.9 ± 3.7	2.4 ± 4.1	1.9 ± 3.0	0.84^a^
Colonoscopy Indication n (%)					0.32^d^
Screening Diagnostic	90 (88.2) 12 (11.8)	37 (84.1) 7 (15.9)	18 (85.7) 3 (14.3)	35 (94.6) 2 (5.4)	
No. patients with Hyperplastic polyps, n (%)	13 (12.6)	4 (9.1)	6 (28.6)	3 (7.9)	0.079^d^
Adenoma detection rate, n (%)	37 (35.9)	6 (13.6)	11 (52.4)	20 (52.6)	***< 0.001*** ^***b***^
Advanced adenoma, n (%)					*N/A*
Size > 10 mm	2 (1.9)	1 (2.3)	1 (4.8)	0 (0.00)	
HGD	1 (0.97)	0 (0.00)	1 (4.8)	0 (0.00)	
Advanced Adenoma detection rate, n (%)	3 (2.9)	1 (2.3)	2 (9.5)	0 (0.00)	0.15^d^
SSL detection rate, n (%)	10 (9.7)	4 (9.1)	1 (4.8)	5 (13.2)	0.69^d^
Advanced SSL detection rate, n (%)	4 (3.9)	2 (4.5)	0 (0.00)	2 (5.3)	0.83^d^
Colorectal cancer, n (%)	3 (2.9)	1 (2.3)	1 (4.8)	1 (2.6)	0.80^d^

Statistics presented as Mean ± SD, Median [P25, P75], N (column %)

p-values: a = ANOVA, b = Kruskal-Wallis test, c = Pearson’s chi-square test, d = Fisher’s Exact test. N/A = not applicable

1: Significantly different from < 45 years

Post-hoc pairwise comparisons were done using Bonferroni adjustment

Demographic and colonoscopy findings were compared between the 91 patients who underwent screening versus the 12 who underwent diagnostic colonoscopy (Supplementary Tables 1 and 2). The only difference observed was a younger median age at time of first colonoscopy in the diagnostic group, 31.8 years vs. 47.0 years in the screening group. (Supplementary Table 2). An analysis of demographic and colonoscopy findings by age range in the screening colonoscopy cohort (Supplementary Tables 3 and 4). showed no substantial differences when compared to our overall cohort results stratified by indication for colonoscopy (Tables 1 and 2). The exception included an association between age and prevalence of breast cancer (Supplementary Table 3). We continued to observe a significant difference in ADR by age: under 45 years (13.5%, 95% CI: 5.91-28.0%), 45–49 years (44.4%, 95% CI:24.6–66.3%), and ≥ 50 years (52.8%, 95% CI:37.0–68.0%), respectively. (Supplementary Table 4).

## Discussion

This is the first international study to investigate colonoscopy findings in *CDH1* PV patients. Our cohort, primarily consisting of female, Caucasian *CDH1* PV carriers, represents the largest assembled group undergoing screening examinations. The overall adenoma detection rate (ADR) was 35.9% (95% CI:27.3–45.5%), with surveillance being the indication in most cases (88.2%), representing the first pan-continental data on ADR in *CDH1* PV carriers.

There were differences in the ADR between Cleveland (48.6%, 95% CI: 33.0-64.4%) and the German cohort (29.4%, 95% CI: 19.9–41.1%), which could be due to various factors; however, this difference was not statistically significant (*p* = 0.055). The ADR in Cleveland (48.6%, 95% CI: 33.0-64.4%) exceeded the U.S. average ADR (35.6%), although this difference was not statistically significant (p-value = 0.08) [11, 12]. The ADR in the German cohort (29.4%, 95% CI: 19.9–41.1%) was similar to the national German average risk screening cohort, which had an overall ADR of 31.3% in men (p-value = 0.84), with an ADR of 20.1% in women (p-value = 0.08), respectively [13]. The possible increased risk of CRC in this population is consistent with the occurrence of early-onset CRC in 2 of the 3 *CDH1* PV carriers diagnosed and the detection of advanced adenomas/SSLs in the age group below 45 years.

A variety of highly penetrant germline PVs are associated with a substantially heightened risk of CRC including those that cause the adenomatous and hamartomatous polyposis syndromes and Lynch syndrome, and these syndromes account for less than 5% of CRC cases [14]. Patients with *CDH1* PV carriers are not targeted as a group with an elevated risk of CRC, yet *CDH1* PV enrichment has recently been detected in individuals with CRC and colorectal signet-ring cell cancer [5, 15].

Surveillance recommendations must always be risk-stratified. Thus, there are clear guidelines for patients with a confirmed genetic predisposition to CRC, as well as for individuals with a positive family history of CRC. ADR is a significant predictor of CRC risk, as multiple studies have demonstrated. In a prospective study conducted in Germany on colonoscopy in individuals with a positive family history of CRC, an ADR of 13.8% was observed in the age group of 25–40 years, and an ADR of 21.2% in those aged 41–50 years. This is comparable to the regular screening cohort in Germany aged 55–59 years, where an ADR of 18.2% was found [16]. In our study, we demonstrated an ADR of 13.5% (95% CI: 5.91-28.0%) in individuals under 45 years, which is similar to the ADR in patients aged 25–40 years with a family history of CRC. Given the limited evidence that *CDH1* PV carriers are more likely to develop neoplasia, it is essential to examine the incidence of preneoplastic lesions and the risk of developing invasive CRC in this population.

Recent studies, such as the one by Ryan et al., have indicated that the risk of diffuse gastric cancer in *CDH1* PV carriers may not be as high as previously thought, making it increasingly important to consider other potentially preventable cancer risks, such as colorectal cancer, in this population [4]. So far, two studies from the United States have analysed pathological findings during colonoscopies in *CDH1* patients. Passi et al. conducted a monocentric study and reported that 47% of 85 *CDH1* PV carriers had at least one adenoma or sessile serrated lesion, with 56.3% of patients being under 45 years old [8]. Stanich et al. conducted a multicentric study, but only within the USA, and reported an ADR of 35.3% (30/85) in *CDH1* PV carriers, with 26.6% of patients (8/30) under 45 years old [9]. No CRC cases were reported in either cohort study. In contrast, our study includes more patients and provides data from an international cohort, highlighting differences between populations. Additionally, we observed three cases of CRC, including two early-onset CRC (EOCRC), which underscores the potential CRC risk in this population.

Within our combined screening data across three age categories, the ADR progressively rose, from 13.5% (95% CI: 5.91-28.0%) in the < 45 years group, to 44.4% (95% CI: 24.6–66.3%) in the 45–49 years group, reaching 52.8% (95% CI: 37.0–68.0%) in patients ≥ 50 years. This is higher than the ADR in the U.S. population of 28.4% in the 45–49 age group (p-value = 0.41) and 35.6% in the 50–75 age group (p-value = 0.12), respectively [11, 12].

In the German cohort, we identified three AAs, resulting in an AA detection rate of 4.4% for this group and 2.9% across the aggregated dataset. This AA rate is comparable to individuals with a familial risk of CRC in Germany, as the FARKOR study reported an AA rate of 5.9% (p-value = 0.8) [16]. This again highlights the elevated risk compared to the general population. The average age of patients with AA in our study was 45 years [IQR 36.0, 46.0], an age where surveillance colonoscopy is not recommended in Germany. Stanich et al. noted that the detection rate of AA in *CDH1* PV carriers under 45 years was 6.1%, further supporting the elevated risk seen in our cohort [9].

Besides the classic adenoma-carcinoma pathway, serrated lesions are also an important risk lesion for colorectal cancer [17]. In our study, 10 patients (9.7%) were found to have sessile serrated lesions (SSLs), among which 4 patients (3.2%) exhibited advanced SSLs measuring ≥ 10 mm. These incidences exceed the general population’s SSL detection rate of 2% and surpass the results from Stanich et al., who reported an SSL detection rate of 5.9% (p-value = 0.08) and an advanced SSL detection rate of 1.2% (p-value = 0.036) [9, 18].

In our international cohort of 103 *CDH1* PV carriers, we identified CRC in 3% of our patients with 2 of the three patients being under the age of 50 years, including a 29-year-old. While previous reports of CRC associated with *CDH1* PV have shown a positive family history, none of our three patients had a family history of CRC [14, 19, 20].

Currently, the United States Multi-Society Task Force on Colorectal Cancer recommends starting regular screening at age 45 as the key to preventing CRC, based on the disease burden among individuals under age 50 [21]. However, these recommendations do not apply to Germany, where routine screening still begins at age 50 [22]. Early screening is recommended for patients with risk factors, especially family history of CRC or germline carriers of specific PVs. In our study, we demonstrated a higher than average ADR in the 45–49 years group and the ≥ 50 years group compared to the U.S. national average but the difference was not statistically significant. Additionally, for individuals under 45 years, we observed an ADR comparable to those with a familial risk of CRC in Germany, emphasizing the elevated risk in this population. We also observed early-onset CRC in 2% of patients of our *CDH1* PV carriers. These finding suggest *CDH1* PV carriers are a risk group benefitting from earlier than average age of onset screening.

The limitations of our study include the retrospective design and the small sample size, even though this is the largest study that has been conducted so far. Another limitation of the study was the lack of case-control design where non-carriers were compared with *CDH1* PV carriers. Despite the above limitations, we were able to include an international cohort of patients, the adenoma detection rate was above the country averages, and detected three cases of CRC in *CDH1* PV carriers.

Guidelines based on limited data recommend initiating CRC screening for *CDH1* PV carriers at age 40, especially if CRC has been documented in family members who are *CDH1* PV carriers [23]. With an ADR of 13.6% (95% CI: 6.40–26.7%) in those under 45 years, our study shows that adenoma formation and thus the risk of CRC begins before the regular screening age in the USA (45 years) and well before the regular screening age in Germany (50 years). Furthermore, it resembles the data for patients at risk for familial CRC, where an ADR of 17.6% is documented [15]. Therefore, our data may contribute to a recommendation for earlier CRC screening, beyond the average-risk population screening guidelines. Larger studies, including prospective surveillance studies that assess the risk of developing colorectal cancer by performing screening colonoscopies in patients with *CDH1* PV are required to confirm our findings.

## Electronic supplementary material

Below is the link to the electronic supplementary material.

Supplementary Material 1

## Data Availability

No datasets were generated or analysed during the current study.
