# Supplementary material for: Colonoscopy findings in CDH1 carriers from a multicenter international study
Source: Fam Cancer. 2025 May 5;24(2):44. doi: 10.1007/s10689-025-00466-8 (PMC12052908; doi:10.1007/s10689-025-00466-8)
Supplement: Supplementary file 1 — Supplementary Material 1 [file 10689_2025_466_MOESM1_ESM.docx]

| **Supplementary Table 1. Demographic characteristics stratified by indication for colonoscopy** | | | | | | | |
| --- | --- | --- | --- | --- | --- | --- | --- |
|  | **Overall (N=103)** | | **Screening (N=91)** | | **Diagnostic (N=12)** | |  |
| **Factor** | **N** | **Statistics** | **N** | **Statistics** | **N** | **Statistics** | **p-value** |
| Sex, n (%) | 103 |  | 91 |  | 12 |  | 0.21^d^ |
| Female |  | 68 (66.0) |  | 58 (63.7) |  | 10 (83.3) |  |
| Male |  | 35 (34.0) |  | 33 (36.3) |  | 2 (16.7) |  |
| Ethnicity, n (%) | 102 |  | 90 |  | 12 |  | 0.19^d^ |
| Caucasian |  | 95 (93.1) |  | 85 (94.4) |  | 10 (83.3) |  |
| African American |  | 3 (2.9) |  | 2 (2.2) |  | 1 (8.3) |  |
| Asian |  | 2 (2.0) |  | 1 (1.1) |  | 1 (8.3) |  |
| Other |  | 2 (2.0) |  | 2 (2.2) |  | 0 (0.00) |  |
| Personal History Gastric Cancer, n (%) | 103 | 50 (48.5) | 91 | 43 (47.3) | 12 | 7 (58.3) | 0.47^c^ |
| Age at Gastric Cancer, yrs, median [Q1, Q3] | 50 | 45.5 [33.0, 53.0] | 43 | 47.0 [34.0, 57.0] | 7 | 38.5 [21.0, 51.0] | 0.12^b^ |
| Personal History Breast Cancer*, n (%) | 68 | 27 (39.7) | 58 | 24 (41.4) | 10 | 3 (30.0) | 0.73^d^ |
| Age at Breast Cancer*, yrs, median [Q1, Q3] | 27 | 48.3 [43.1, 59.0] | 24 | 48.5 [43.4, 59.1] | 3 | 45.0 [35.5, 49.0] | 0.29^b^ |
| Family History Colorectal Cancer, n (%) | 103 | 23 (22.3) | 91 | 19 (20.9) | 12 | 4 (33.3) | 0.46^d^ |
| Age of Relative at CRC, yrs, median [Q1, Q3] | 2 | 61.5 [60.0, 63.0] | 2 | 61.5 [60.0, 63.0] | 0 | *Not available* | ***N/A*** |
| * = Females only; Statistics presented as Median [P25, P75], N (column %). p-values: b=Wilcoxon Rank Sum test, c=Pearson's chi-square test, d=Fisher's Exact test. | | | | | | | |

| **Supplementary Table 2. Colonoscopy findings stratified by colonoscopy indication** | | | | | | | |
| --- | --- | --- | --- | --- | --- | --- | --- |
|  | **Overall (N=103)** | | **Screening (N=91)** | | **Diagnostic (N=12)** | |  |
| **Factor** | **N** | **Statistics** | **N** | **Statistics** | **N** | **Statistics** | **p-value** |
| Age at 1st colonoscopy, yrs, median, [Q1, Q3] | 103 | 47.0 [34.5, 56.0] | 91 | 47.0 [36.0, 56.0] | 12 | 31.8 [18.3, 48.0] | ***0.025^b^*** |
| Age at first colonoscopy, n (%) | 103 |  | 91 |  | 12 |  | 0.15^b^ |
| <45 years |  | 44 (42.7) |  | 37 (40.7) |  | 7 (58.3) |  |
| 45-49 years |  | 21 (20.4) |  | 18 (19.8) |  | 3 (25.0) |  |
| ≥ 50 |  | 38 (36.9) |  | 36 (39.6) |  | 2 (16.7) |  |
| Number of colonoscopies, median [Q1, Q3] | 103 | 1.00 [1.00, 2.0] | 91 | 1.00 [1.00, 2.0] | 12 | 1.5 [1.00, 3.0] | 0.28^b^ |
| Number of colonoscopies, n (%) | 103 |  | 91 |  | 12 |  | 0.17^d^ |
| 1 |  | 62 (60.2) |  | 56 (61.5) |  | 6 (50.0) |  |
| 2 |  | 25 (24.3) |  | 23 (25.3) |  | 2 (16.7) |  |
| 3 |  | 9 (8.7) |  | 6 (6.6) |  | 3 (25.0) |  |
| ≥ 4 |  | 7 (6.8) |  | 6 (6.6) |  | 1 (8.3) |  |
| Years of Colonoscopy follow up, mean ± sd | 103 | 2.0 ± 3.5 | 91 | 1.8 ± 3.0 | 12 | 3.3 ± 6.2 | 0.44^a2^ |
| Hyperplastic Polyp, n (%) | 103 | 13 (12.6) | 91 | 11 (12.1) | 12 | 2 (16.7) | 0.65^d^ |
| Age at 1st non-advanced adenoma, yrs, median, [Q1, Q3] | 37 | 53.0 [47.0, 59.0] | 32 | 55.0 [47.0, 59.5] | 5 | 47.9[47.9, 49.8] | 0.39^b^ |
| Adenoma detection rate, n (%) | 103 | 37 (35.9) | 91 | 32 (35.2) | 12 | 5 (41.7) | 0.75^d^ |
| Advanced Adenoma detection rate, n (%) | 103 | 3 (2.9) | 91 | 3 (3.3) | 12 | 0 (0.00) | 0.99^d^ |
| Age at first Advanced Adenoma, median [Q1, Q3] | 3 | 45.0 [36.0, 46.0] | 3 | 45.0 [36.0, 46.0] | 0 | --- | N/A |
| SSL detection rate, n (%) | 103 | 10 (9.7) | 91 | 9 (9.9) | 12 | 1 (8.3) | 0.99^d^ |
| Advanced SSL (>10 mm or dysplasia), n (%) | 103 | 4 (3.9) | 91 | 3 (3.3) | 12 | 1 (8.3) | 0.40^d^ |
| CRC, n (%) | 103 | 3 (2.9) | 91 | 2 (2.2) | 12 | 1 (8.3) | 0.31^d^ |
| Statistics presented as Mean ± SD, Median [P25, P75], N (column %). p-values: a1=t-test, a2=Satterthwaite t-test, b=Wilcoxon Rank Sum test, d=Fisher's Exact test. | | | | | | | |

| **Supplementary Table 3. Demographic characteristics of patients with “screening” indication for colonoscopy stratified by age** | | | | | | | | | |
| --- | --- | --- | --- | --- | --- | --- | --- | --- | --- |
|  | **Overall (N=91)** | | **<45 years (N=37)** | | **45-49 years (N=18)** | | **≥ 50 (N=36)** | |  |
| **Factor** | **N** | **Statistics** | **N** | **Statistics** | **N** | **Statistics** | **N** | **Statistics** | **p-value** |
| Sex, n (%) | 91 |  | 37 |  | 18 |  | 36 |  | 0.65^c^ |
| Female |  | 58 (63.7) |  | 22 (59.5) |  | 13 (72.2) |  | 23 (63.9) |  |
| Male |  | 33 (36.3) |  | 15 (40.5) |  | 5 (27.8) |  | 13 (36.1) |  |
| Ethnicity, n (%) | 90 |  | 37 |  | 17 |  | 36 |  | 0.39^d^ |
| Caucasian |  | 85 (94.4) |  | 36 (97.3) |  | 16 (94.1) |  | 33 (91.7) |  |
| African American |  | 2 (2.2) |  | 0 (0.00) |  | 0 (0.00) |  | 2 (5.6) |  |
| Asian |  | 1 (1.1) |  | 0 (0.00) |  | 1 (5.9) |  | 0 (0.00) |  |
| Other |  | 2 (2.2) |  | 1 (2.7) |  | 0 (0.00) |  | 1 (2.8) |  |
| Personal History Gastric Cancer, n (%) | 91 | 43 (47.3) | 37 | 20 (54.1) | 18 | 7 (38.9) | 36 | 16 (44.4) | 0.52^c^ |
| Age at Gastric Cancer, yrs, median, [Q1, Q3] | 43 | 47.0 [34.0, 57.0] | 20 | 33.5 [28.0, 40.5] ^2,3^ | 7 | 48.5 [47.0, 50.0] ^1,3^ | 16 | 59.0 [53.0, 62.1] ^1,2^ | ***<0.001^b^*** |
| Personal History Breast Cancer*, n (%) | 58 | 24 (41.4) | 22 | 5 (22.7) ^3^ | 13 | 5 (38.5) | 23 | 14 (60.9) ^1^ | ***0.033^c^*** |
| Age at Breast Cancer*, yrs, median [Q1, Q3] | 24 | 48.5 [43.4, 59.1] | 5 | 40.0 [38.9, 42.0] ^2,3^ | 5 | 46.0 [46.0, 46.1] ^1,3^ | 14 | 58.0 [53.0, 61.7] ^1,2^ | ***0.001^b^*** |
| Family History Colorectal Cancer, n (%) | 91 | 19 (20.9) | 37 | 8 (21.6) | 18 | 4 (22.2) | 36 | 7 (19.4) | 0.96^c^ |
| Age of relative with Colorectal Cancer, yrs, median [Q1, Q3] | 2 | 61.5 [60.0, 63.0] | 1 | 63.0 [63.0, 63.0] | 1 | 60.0 [60.0, 60.0] | 0 | *Not available* | ***N/A*** |
| * = females; Statistics presented as Median [P25, P75], N (column %). p-values: b=Kruskal-Wallis test, c=Pearson's chi-square test, d=Fisher's Exact test. ^1^: Significantly different from <45 years ^2^: Significantly different from 45-49 years ^3^: Significantly different from ≥ 50 Post-hoc pairwise comparisons were done using Bonferroni adjustment. | | | | | | | | | |

| **Supplementary Table 4. Screening colonoscopy findings stratified by age range at first screening colonoscopy** | | | | | | | | | |
| --- | --- | --- | --- | --- | --- | --- | --- | --- | --- |
|  | **Overall (N=91)** | | **<45 years (N=37)** | | **45-49 years (N=18)** | | **≥ 50 (N=36)** | |  |
| **Factor** | **N** | **Statistics** | **N** | **Statistics** | **N** | **Statistics** | **N** | **Statistics** | **p-value** |
| Age at 1st colonoscopy, yrs, median [Q1, Q3] | 91 | 47.0 [36.0, 56.0] | 37 | 34.0 [31.0, 39.0] | 18 | 47.3 [46.2, 49.2] | 36 | 57.8 [55.5, 61.5] | ***<0.001^b^*** |
| Number of colonoscopies, median [Q1, Q3] | 91 | 1.00 [1.00, 2.0] | 37 | 1.00 [1.00, 2.0] | 18 | 1.00 [1.00, 2.0] | 36 | 1.00 [1.00, 2.0] | 0.69^b^ |
| Total number of colonoscopies, n (%) | 91 |  | 37 |  | 18 |  | 36 |  | 0.97^d^ |
| 1 |  | 56 (61.5) |  | 24 (64.9) |  | 12 (66.7) |  | 20 (55.6) |  |
| 2 |  | 23 (25.3) |  | 8 (21.6) |  | 4 (22.2) |  | 11 (30.6) |  |
| 3 |  | 6 (6.6) |  | 3 (8.1) |  | 1 (5.6) |  | 2 (5.6) |  |
| ≥ 4 |  | 6 (6.6) |  | 2 (5.4) |  | 1 (5.6) |  | 3 (8.3) |  |
| Years of colonoscopy follow-up, median [Q1, Q3] | 91 | 0.00 [0.00, 4.0] | 37 | 0.00 [0.00, 4.0] | 18 | 0.00 [0.00, 3.8] | 36 | 0.00 [0.00, 4.3] | 0.90^b^ |
| Hyperplastic Polyp, n (%) | 91 | 11 (12.1) | 37 | 4 (10.8) | 18 | 5 (27.8) | 36 | 2 (5.6) | 0.078^d^ |
| Adenoma detection rate, n (%) | 91 | 32 (35.2) | 37 | 5 (13.5) | 18 | 8 (44.4) | 36 | 19 (52.8) | ***0.001^c^*** |
| Age at 1st non-advanced adenoma, yrs, median [Q1, Q3] | 32 | 55.0 [47.0, 59.5] | 5 | 39.0 [35.1, 42.0] | 8 | 47.0 [46.1, 49.1] | 19 | 58.0 [56.0, 63.0] | ***<0.001^b^*** |
| SSL detection rate, n (%) | 91 | 9 (9.9) | 37 | 3 (8.1) | 18 | 1 (5.6) | 36 | 5 (13.9) | 0.66^d^ |
| Advanced Adenoma detection rate, n (%) | 91 | 3 (3.3) | 37 | 1 (2.7) | 18 | 2 (11.1) | 36 | 0 (0.00) | 0.099^d^ |
| Age at first Advanced Adenoma, yrs, median [Q1, Q3] | 3 | 45.0 [36.0, 46.0] | 1 | 36.0 [36.0, 36.0] | 2 | 45.5 [45.0, 46.0] | 0 | --- | 0.22^b^ |
| Advanced SSL (>10 mm or dysplasia), n (%) | 91 | 3 (3.3) | 37 | 1 (2.7) | 18 | 0 (0.00) | 36 | 2 (5.6) | 0.61^d^ |
| CRC, n (%) | 91 | 2 (2.2) | 37 | 0 (0.00) | 18 | 1 (5.6) | 36 | 1 (2.8) | 0.35^d^ |
| Statistics presented as Median [P25, P75], N (column %). p-values: b=Kruskal-Wallis test, c=Pearson's chi-square test, d=Fisher's Exact test. | | | | | | | | | |
